# Integrated transcriptome and metabolome analysis reveals key genes and metabolic pathways regulating growth and development in *Polygonatum cyrtonema* Hua

**DOI:** 10.3389/fpls.2026.1940943

**Published:** 2026-09-03

**Authors:** Yu Wang, Haiyang Zhao, Wenjie He, Yijun Ke, Rui Ma, Can Huang, Liang Wu, Lubo Hua

**Affiliations:** 1 Department of Pharmacy, Zhejiang Hospital, Hangzhou, Zhejiang, China; 2 Institute of Life Sciences, Biomedical Collaborative Innovation Center of Zhejiang Province, Wenzhou University, Wenzhou, China; 3 Department of Pharmacy, Anqing Municipal Hospital, Anqing, China

**Keywords:** *Polygonatum cyrtonema* Hua, transcriptome, metabolome, growth mechanism, differentially expressed genes (DEGs)

## Abstract

*Polygonatum cyrtonema* Hua is a traditional Chinese medicine with the same origin as both medicine and food, and its medicinal components have considerable clinical value. Due to its substantial market demand, it is now primarily produced through artificial cultivation. To produce high-quality *P. cyrtonema*, we performed transcriptome and metabolome sequencing of one-year-old and three-year-old *P. cyrtonema* to explore the growth regulation mechanisms and key genes involved in improving its quality. A total of 1,957 differentially expressed genes (DEGs) and 163 differentially expressed metabolites (DEMs) were identified in this study. Integrated transcriptomic and metabolomic analyses suggested that the growth regulation of *P. cyrtonema* may be primarily associated with sphingolipid metabolism, phenylpropanoid biosynthesis, and starch and sucrose metabolism. Our data suggest that sucrose transport to sink organs may be facilitated by increased expression of the bidirectional sugar transporter *SWEET14*, and sucrose may be hydrolyzed by *β-fructofuranosidase*, potentially providing energy for plant growth on one hand and contributing to fructose accumulation on the other. Furthermore, the elevated abundance of L-phenylalanine may be associated with an increase in secondary metabolites, which could provide a metabolic basis for age-dependent growth and metabolite partitioning in rhizomes. The observed downregulation of sphingolipid metabolism-related genes may reflect the perennial growth habit of *P. cyrtonema*, whereby slower growth in the first year may promote sphingolipid-mediated root development. However, we emphasize that these inferences are based on correlative transcriptomic and metabolomic data, and functional validation is required to establish causal relationships.

## 1 Introduction

*Polygonatum cyrtonema* Hua is a perennial herb of the family Asparagaceae, and its medicinal part is the rhizome. It is one of the traditional Chinese medicines that can be used as both medicine and food (Feng et al., 2020; Qi et al., 2022). *P. cyrtonema* contains numerous bioactive natural products, including polysaccharides, steroidal saponins, and flavonoids (Li et al., 2022; Han et al., 2023; Zhang et al., 2024). The pharmacological effects of its chemical constituents have been extensively investigated: polysaccharides from *P. cyrtonema* have demonstrated antioxidant and antimicrobial activities (Li et al., 2018b), anti-inflammatory effects (He et al., 2021), hepatoprotective properties (Liu et al., 2022), regulation of atherosclerosis (Guo et al., 2024), anti-fatigue activity (Shen et al., 2021), modulation of gut microbiota (Xu et al., 2023), amelioration of chemotherapy-induced cachexia (Tang et al., 2023), anti-depressant effects (Shen et al., 2022), and immunomodulatory functions (Xie et al., 2024). These diverse bioactivities have spurred broad application prospects for *P. cyrtonema*.

However, with the continuous development of new products in the market, the supply of wild *P. cyrtonema* no longer meets the increasing demand. Moreover, the natural habitats of *P. cyrtonema* have been degraded due to land development, and most *P. cyrtonema* is now produced through artificial cultivation (Mengdi et al., 2021). Yet the quality and efficacy of cultivated medicinal materials are often inferior to those of their wild counterparts. Consequently, in addition to research on processing methods, extraction and isolation of active components, pharmacology, and clinical applications, current efforts have mainly focused on simulating wild growing environments, tissue culture, and breeding (Li et al., 2018a; He et al., 2021; Hu et al., 2022; Zhang et al., 2022). Relatively few studies have applied molecular biology approaches to enhance the germplasm resources of *P. cyrtonema* based on an understanding of its growth regulatory mechanisms.

Previous studies have demonstrated that the content of bioactive components such as polysaccharides and saponins in *P. cyrtonema* varies with plant age; therefore, to ensure medicinal quality, *P. cyrtonema* is typically harvested at 3–5 years of age (Pu et al., 2024). To systematically investigate the key mechanisms and genes potentially regulating the growth and quality formation of *P. cyrtonema*, we performed an integrated transcriptomic and metabolomic analysis. Our study was designed based on the plant “source–sink” paradigm: we profiled the transcriptomes of leaves (as the source organs for photosynthesis and precursor synthesis, e.g., sucrose and phenylalanine) and the metabolomes of rhizomes (as the primary sink organs for the storage and transformation of medicinal compounds). This source–sink paired approach allows us to identify leaf-expressed genes whose expression patterns are correlated with the accumulation of key metabolites in the rhizome across different growth years. Subsequently, differentially expressed genes (DEGs) and differentially expressed metabolites (DEMs) were analyzed via Gene Ontology (GO) and Kyoto Encyclopedia of Genes and Genomes (KEGG) enrichment analyses to identify metabolic pathways and candidate genes potentially associated with growth and development, thereby providing insights and genetic resources for the improvement of *P. cyrtonema* germplasm. It is important to note that all gene–metabolite associations reported in this study are correlative in nature; functional validation studies will be required to establish causal relationships.

## 2 Materials and methods

### 2.1 Plants source and treatment

The one-year-old and three-year-old *P. cyrtonema* plants used in this study were cultivated at the Plant Research Base of Chizhou, Anhui Province, China. The plants were propagated from seeds obtained from a local, genetically stable wild population in the same ecological region. They were grown under standardized cultivation conditions, including regulated irrigation, fertilization, and pest control management. Sampling was conducted in mid-May. For transcriptome analysis, fully expanded healthy leaves from the middle section of the stem were collected. For metabolomic analysis, mature rhizome segments from the same plants were harvested. After a two-day acclimation period under laboratory conditions, the collected plant materials were thoroughly washed with pure water, rinsed with phosphate-buffered saline (PBS), and gently blotted dry. The required tissues were then immediately frozen in liquid nitrogen and stored at −80 °C until further analysis. Three biological replicates were prepared for each group.

### 2.2 RNA extraction and sequence analysis

Total RNA was extracted from leaf samples using the E.Z.N.A. Plant RNA Kit (Omega Bio-tek, USA) according to the manufacturer’s instructions. RNA quality was assessed using an Agilent 2100 Bioanalyzer, and high-quality RNA samples (RNA integrity number ≥ 7.0) were used for library construction. The mRNA was enriched from total RNA using Oligo(dT) magnetic beads and then fragmented into fragments of approximately 300 bp. Using the fragmented mRNA as a template, first-strand cDNA was synthesized with random hexamer primers, followed by second-strand cDNA synthesis. The double-stranded cDNA fragments were purified, end-repaired, A-tailed, and ligated to sequencing adapters. After PCR amplification, the final sequencing libraries were constructed. Library quality was evaluated using an Agilent 2100 Bioanalyzer. Paired-end sequencing (150 bp × 2) was performed on the Illumina sequencing platform.

The raw sequencing data were processed to remove adapters and low-quality reads (Q < 20) using fastp (v0.23.2). The resulting high-quality clean reads were assembled *de novo* into transcripts using Trinity (v2.15.1). Based on the De Bruijn Graph (DBG) algorithm, the high-quality reads were assembled *de novo* into 164,565 transcripts, with a mean length of 1,092 bp. These transcripts were then clustered, and the longest transcript from each cluster was defined as a unigene. All unigenes were functionally annotated by aligning them against six public databases: NR, GO, KEGG, Swiss-Prot, eggNOG, and Pfam. Gene expression levels were quantified as RPKM (Reads Per Kilobase per Million mapped reads) using RSEM (v1.3.3). Differential expression analysis between the two groups was performed with DESeq2 (v1.30.1), applying a threshold of |log_2_(fold change)| > 1 and a false discovery rate (FDR) < 0.05 to identify differentially expressed genes (DEGs). Functional enrichment analysis, including GO and KEGG pathway analyses, was conducted on the identified DEGs using the clusterProfiler R package (v4.0.5). Terms with an FDR < 0.05 were considered significantly enriched.

### 2.3 Quantitative real-time PCR analysis

To validate the reliability of the transcriptome sequencing data, the expression levels of 10 randomly selected DEGs were verified by quantitative real-time PCR (qRT-PCR). Gene-specific primers were designed using Lasergene PrimerSelect software (v7.0, DNASTAR Inc., USA) following the manufacturer’s guidelines. The amplification efficiency for each primer pair was validated and confirmed to be between 95% and 105%, ensuring comparable efficiencies between reference and target genes. The qRT-PCR reactions were performed using a SYBR Green detection system. Each 20 μL reaction mixture contained 50 ng of cDNA template. The 18S rRNA gene was used as an internal reference to normalize the expression data, and the relative expression levels of the target genes were calculated using the 2^−ΔΔCT^ method (Livak and Schmittgen, 2001). All primer sequences are listed in **Supplementary Table 1**.

### 2.4 Extraction, analysis and identification of metabolites

Fresh rhizome samples (100 mg wet weight) were ground into a fine powder in liquid nitrogen. The powder was passed through an 80-mesh sieve to ensure particle size uniformity across all samples from both groups. The chemical components in the rhizome were extracted with 80% (v/v) methanol. After centrifugation, the supernatant was collected and diluted to 53% (v/v) methanol with chromatographic-grade water. Following a second centrifugation, the supernatant was obtained for LC-MS analysis. Each group had three biological replicates. Equal volumes from each experimental sample were pooled to prepare quality control (QC) samples. A 53% (v/v) methanol aqueous solution was used as a blank control. Both QC and blank samples were analyzed using the same procedure as the experimental samples.

For metabolite separation, ultra-high performance liquid chromatography (UHPLC; Thermo Fisher, Germany) coupled with an Orbitrap Q Exactive™ HF-X mass spectrometer (Thermo Fisher, Germany) was employed. The test solution was injected at a flow rate of 0.2 mL/min onto a Hypersil Gold column (100 × 2.1 mm, 1.9 μm), and the column temperature was maintained at 40 °C. The mobile phase A in the positive ionization mode consisted of water containing 0.1% (v/v) formic acid, while in the negative ionization mode, it consisted of 5 mM ammonium acetate (pH 9.0). The mobile phase B in both modes was methanol. The mass spectrometry parameters were as follows: spray voltage, 3.2 kV; sheath gas flow rate, 40 arbitrary units (arb); auxiliary gas flow rate, 10 arb; and capillary temperature, 320 °C.

The raw data acquired from UHPLC-MS/MS were processed as follows: initial filtering was performed based on retention time and mass-to-charge ratio (m/z), followed by peak alignment within a specified deviation range. Peak detection and integration were then conducted to determine metabolite intensities. Metabolite identification was performed by matching the accurate mass and MS/MS fragmentation spectra against standard references in the mzCloud, mzVault, and Masslist databases. Relative quantification was based on the peak areas obtained from UHPLC analysis. Subsequently, multivariate statistical analyses, including princi*pal* component analysis (PCA) and orthogonal partial least squares-discriminant analysis (OPLS-DA), were performed using the R package gmodels (v2.18.1) in both positive and negative ion modes. Differentially expressed metabolites (DEMs) were identified using the combined criteria of a Student’s t-test p-value < 0.05 and a variable importance in projection (VIP) value ≥ 1 from the OPLS-DA model. This dual criterion, combining univariate statistical testing with multivariate pattern recognition, is widely adopted in non-targeted metabolomics studies and balances statistical significance with biological relevance captured by the multivariate model. Finally, the KEGG database was utilized for pathway enrichment analysis of the identified DEMs.

## 3 Results

### 3.1 Gene changes in *P. cytonema* in different years

#### 3.1.1 Overview

Through transcriptome sequencing of one-year-old (Group A) and three-year-old (Group C) *P. cyrtonema*, a total of 45,306,398 and 44,361,502 raw reads were obtained, respectively. After filtering the raw data, the one-year-old group yielded 40,249,718 clean reads while the three-year-old group yielded 40,671,892 clean reads. The percentages of clean reads relative to raw reads were 88.84% and 91.68%, respectively (**Table 1**). Based on the De Bruijn Graph (DBG) algorithm, the high-quality reads were assembled *de novo* into 164,565 transcripts, with a mean length of 1,092 bp. Unigenes, defined as the longest transcript of each gene cluster, represented the representative sequence for each gene. A total of 69,373 unigenes were obtained, with an average length of 900 bp, an N50 length of 1,286 bp, and an N90 length of 393 bp (**Table 2**). To evaluate the functional annotation coverage of these 69,373 unigenes, gene function annotation was performed. The results showed that the NR database (47.79%) and the eggNOG database (45.94%) provided the highest annotation rates, followed by the Swiss-Prot database (37.80%) and the Pfam database (27.99%). The KEGG database (22.62%) and the GO database (17.85%) had relatively lower annotation rates. A total of 5,453 unigenes (7.86%) were annotated in all six databases (**Table 3**).

**Table 1:** Summary of the sequence analyses.

Sample	Raw reads	Clean reads	Q20(%)	Q30(%)	Clean reads( %)
A	45,306,398	40,249,718	97.15	92.64	88.84
C	44,361,502	40,671,892	97.38	93.11	91.68

Clean reads: The number of reads after removing low-quality sequences. The subsequent analysis is based on clean reads.Q20 and Q30, the percentage of bases with Phred values >20 and >30, respectively.

**Table 2:** Sequence population statistics.

Conting	Transcript	Unigene
Total Length (bp)	179807843	62494832
Sequence Number	164565	69373
Max. Length (bp)	15739	15739
Mean Length (bp)	1092.63	900.85
N50 (bp)	1556	1286
N50 Sequence No.	35608	13739
N90 (bp)	480	393
N90 Sequence No.	117118	51093
GC%	43.60	43.26

Total length (bp): total length of sequence. Sequence number: total number of sequences. Max. length (bp): maximum length of sequence. Mean length: average length of the sequence. N50 (bp): arrange all sequences from long to short, and add the sequence length in this order. When the added length reaches 50% of the total length of the sequence, the length of the last sequence. N90 (bp): arrange all sequences from long to short, and add the sequence length in this order. When the added length reaches 90% of the total length of the sequence, the length of the last sequence. N50 sequence No.: total number of sequences with length greater than N50. N90 sequence No.: total number of sequences with length greater than N90.GC%: GC content of the sequence.

**Table 3:** The success rate of gene annotation.

Database	Number	Percentage
NR	33151	47.79
eggNOG	31870	45.94
Swissprot	26226	37.8
Pfam	19417	27.99
KEGG	15693	22.62
GO	12381	17.85
In all database	5453	7.86

Database: type of database. Number: represents the number of unigenes successfully annotated in the database. Percentage (%): represents the proportion of unigenes successfully annotated in the database to the total unigenes. In all database: the number of unigenes annotated in all databases.

#### 3.1.2 Identification and functional analysis of differentially expressed genes

**Figure 1:**
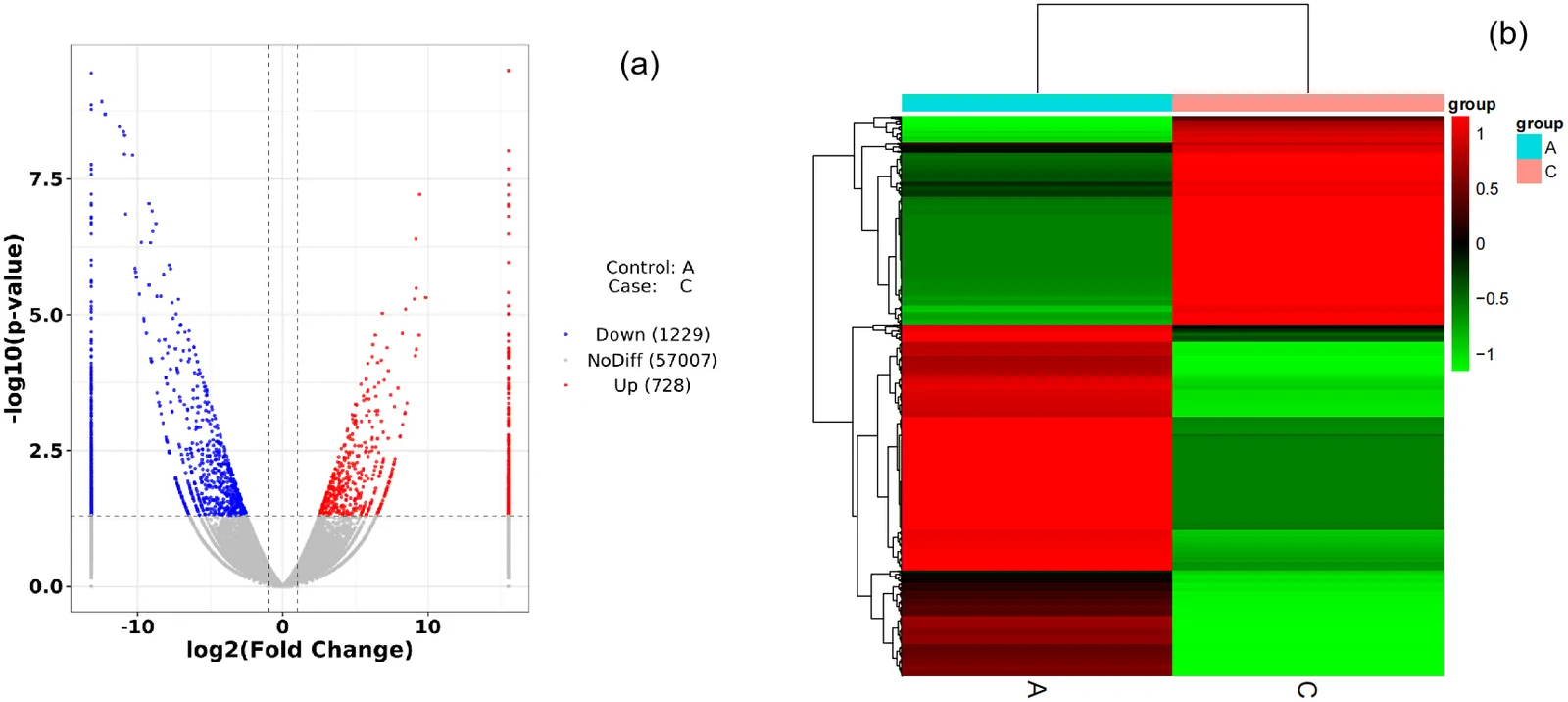
Expression of differential genes in the transcriptome: **(a)** volcano plot of differential genes, **(b)** cluster diagram of differential gene expression patterns.

Gene expression levels were quantified as RPKM, and differentially expressed genes (DEGs) were identified based on fold change and statistical significance between the two groups. According to the screening criteria (|log_2_(fold change)| > 1 and FDR < 0.05), a total of 1,957 DEGs were identified, including 728 up-regulated and 1,229 down-regulated genes (**Figure 1a**). The expression patterns of the two groups of genes showed distinct separation in the correlation cluster heatmap of differential genes (**Figure 1b**).

**Figure 2:**
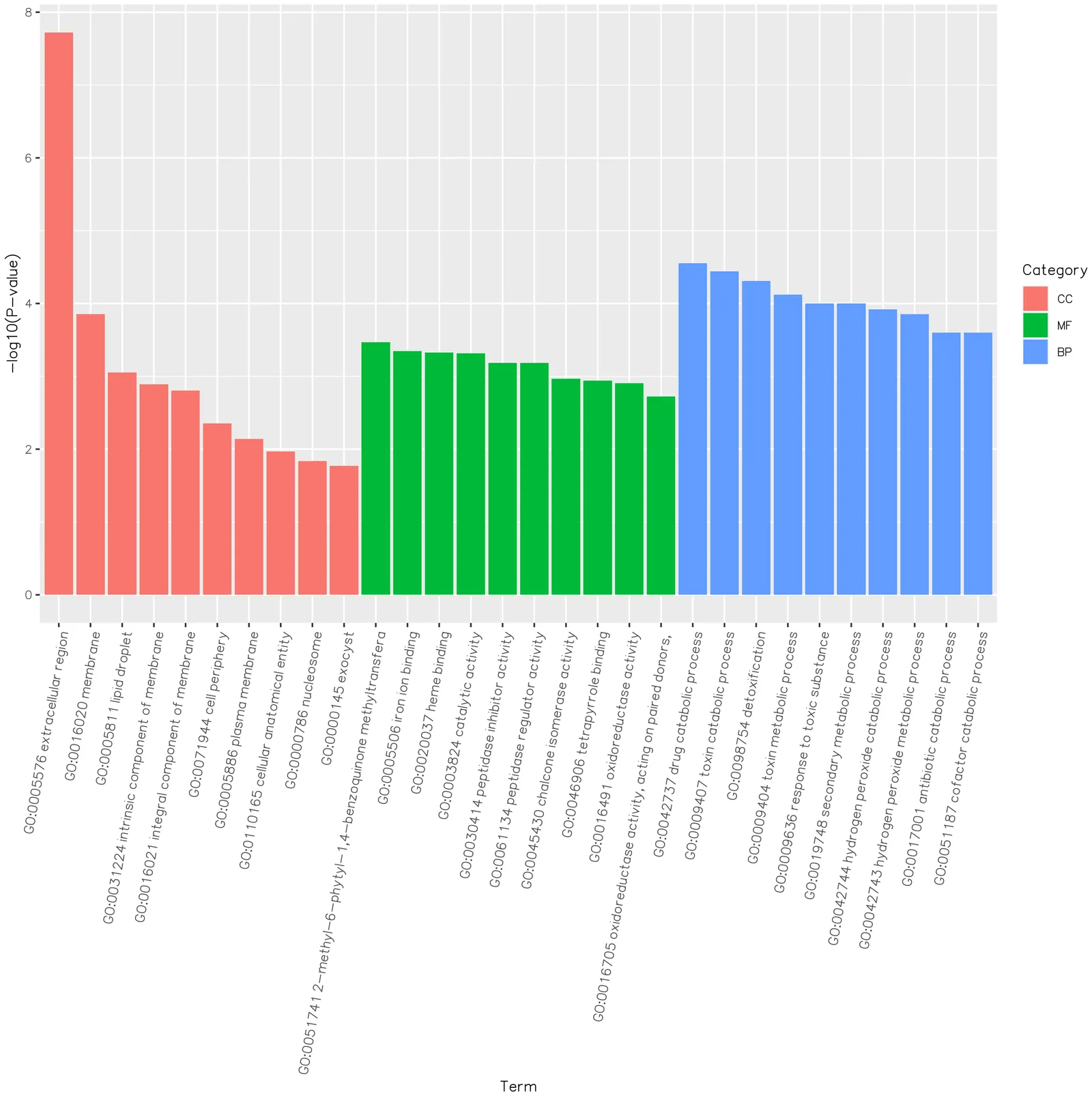
10 terms of each GO classification.

**Figure 3:**
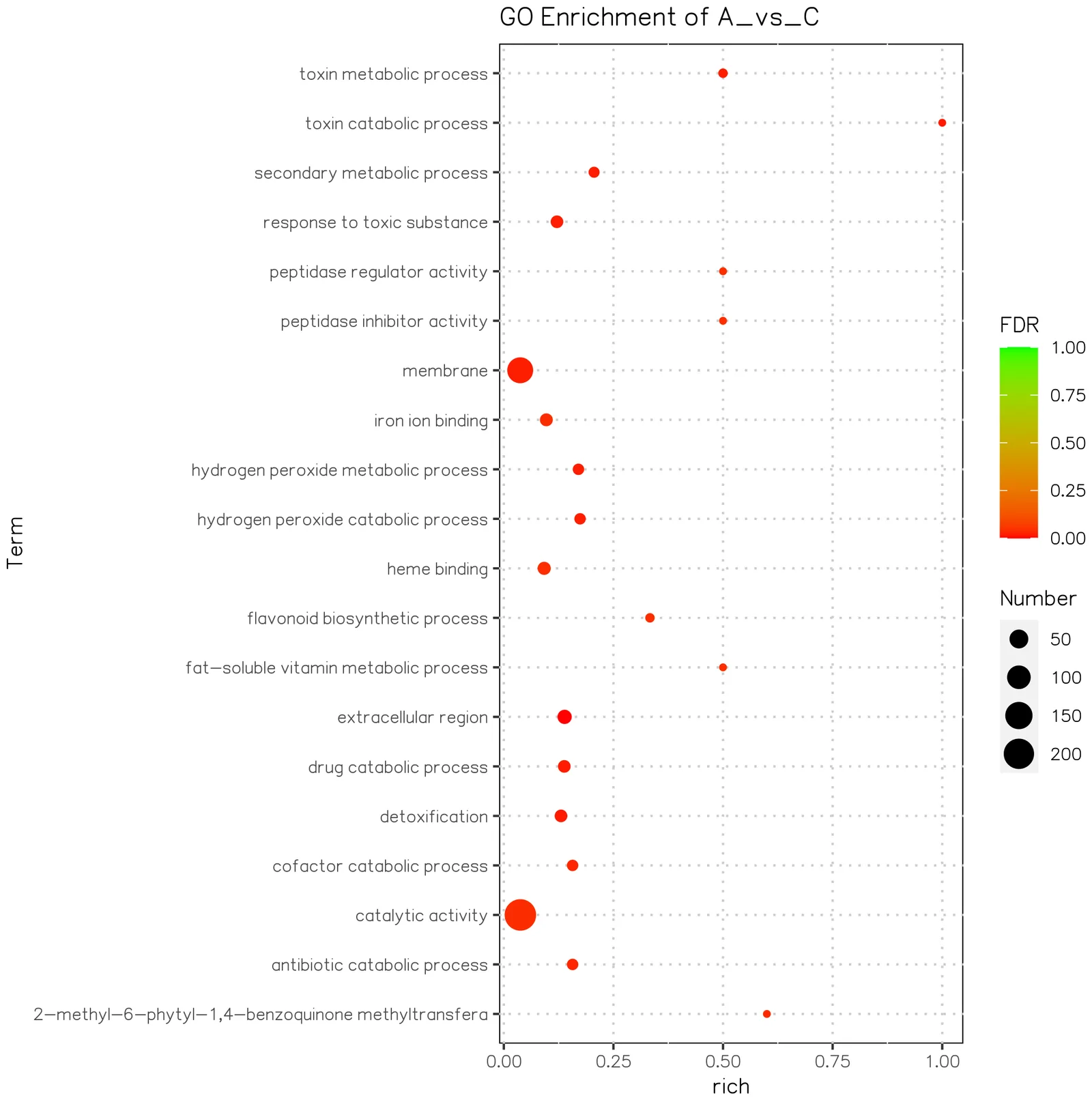
Top 20 terms of GO pathway enrichment results.

The identified DEGs were compared to GO annotation categories within biological processes (BP), molecular functions (MF), and cellular components (CC). Among the top 10 GO terms for each GO classification, the extracellular region was significantly enriched (**Figure 2**). Among the top 20 enriched GO terms, catalytic activity and membrane were the most significantly enriched (**Figure 3**).

**Figure 4:**
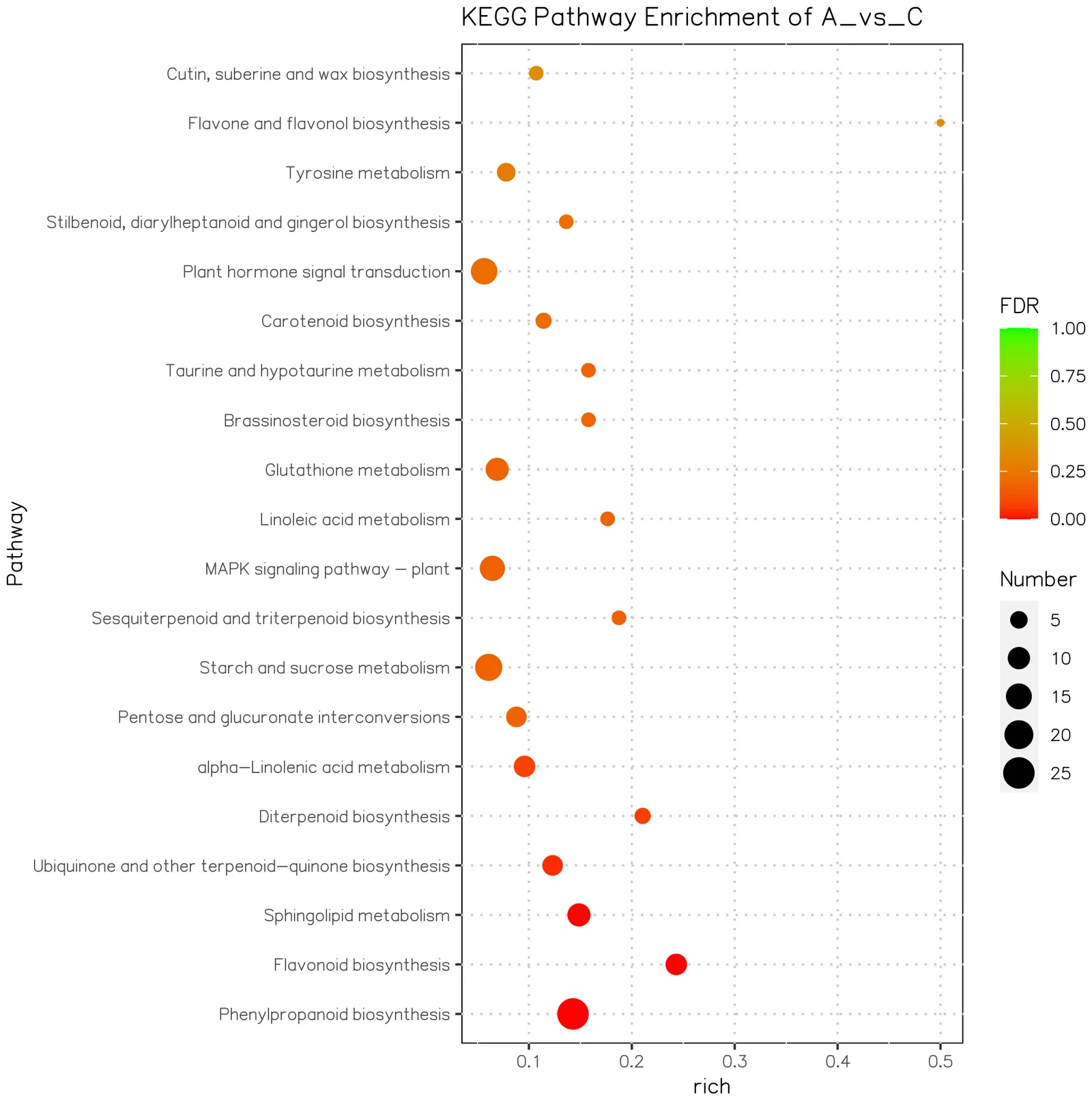
KEGG pathway enrichment analysis of differentially expressed genes among different groups. Bubble charts ranks top 20 KEGG pathways, and the larger bubble size indicates higher number of involved DEGs.

KEGG enrichment analysis of the DEGs revealed their significant involvement in 102 pathways, with a predominant emphasis on metabolic processes. Notably, among the most significantly enriched pathways (**Figure 4**) were phenylpropanoid biosynthesis, starch and sucrose metabolism, and sphingolipid metabolism, suggesting their potential involvement in the age-dependent growth and development of *P. cyrtonema*. Furthermore, genes encoding key enzymes and transporters within these pathways were identified as differentially expressed, including SWEET14 (a bidirectional sugar transporter) and β-fructofuranosidase in the starch and sucrose metabolism pathway, and phenylalanine ammonia-lyase (PAL) in the phenylpropanoid biosynthesis pathway. The identification of these candidate genes provides specific targets for further investigation of the molecular mechanisms potentially underlying growth and metabolite accumulation.

**Figure 5:**
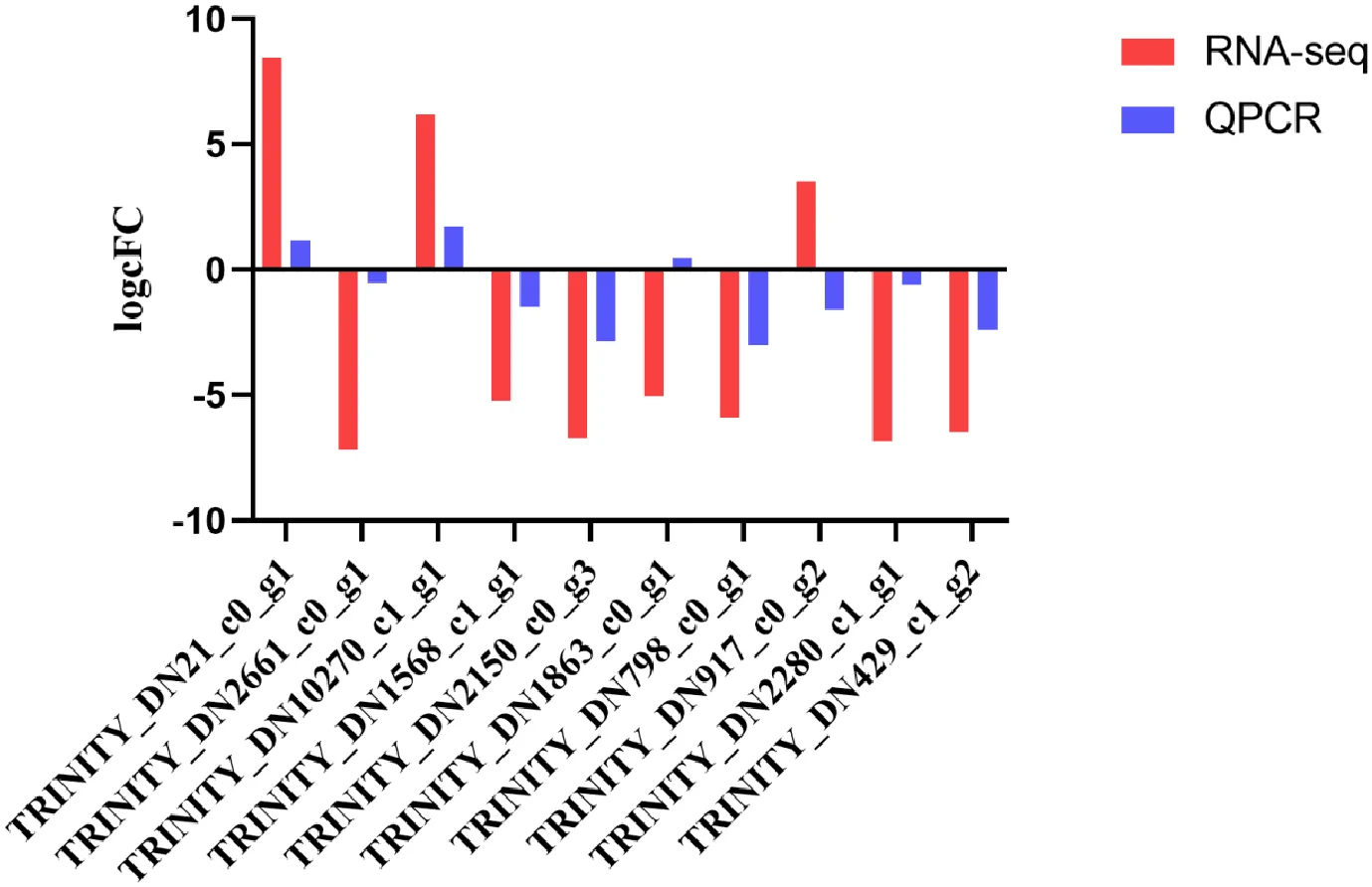
Validation of qPCR: comparison of the relative log_2_Foldchange between RNA-Seq results and qPCR, as normalized with the 18S gene.

To assess the reliability of the transcriptome data, ten genes were selected for qRT-PCR validation. Comparison of the qRT-PCR results with the RNA-Seq data revealed that the expression trends of the majority of genes were consistent with the transcriptome data, indicating that the transcriptome data are reliable (**Figure 5**).

### 3.2 Metabolomic changes in P. cyrtonema across different growth years

#### 3.2.1 Overview of metabolome profiling

**Figure 6:**
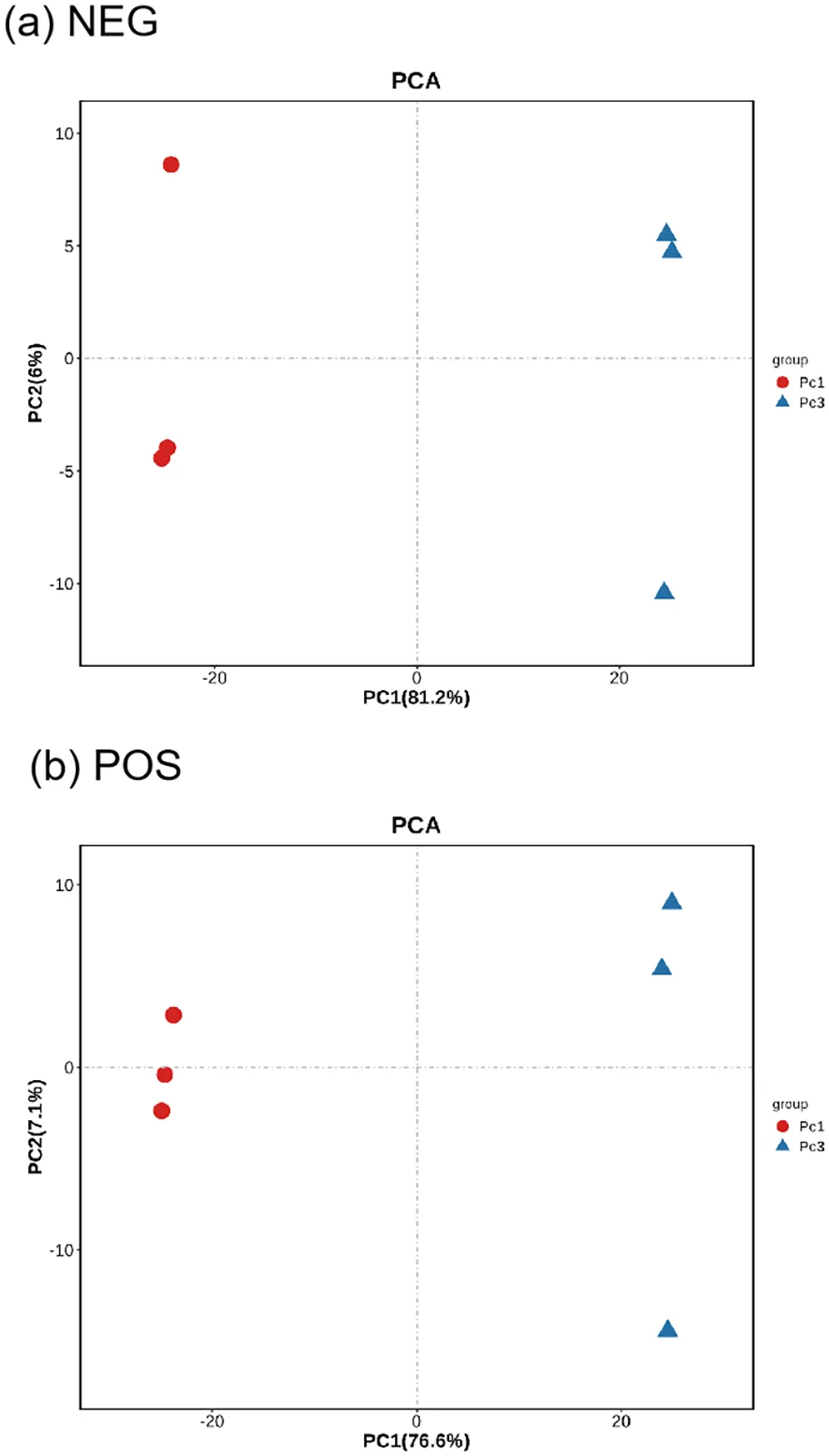
The PCA among different study samples of *P. cyrtonema* **(a)** the negative (ESI^−^) ion mode, **(b)** the positive (ESI+) ion mode. QC refers to the quality control sample, which is made by mixing parts of each analytical sample.

**Figure 7:**
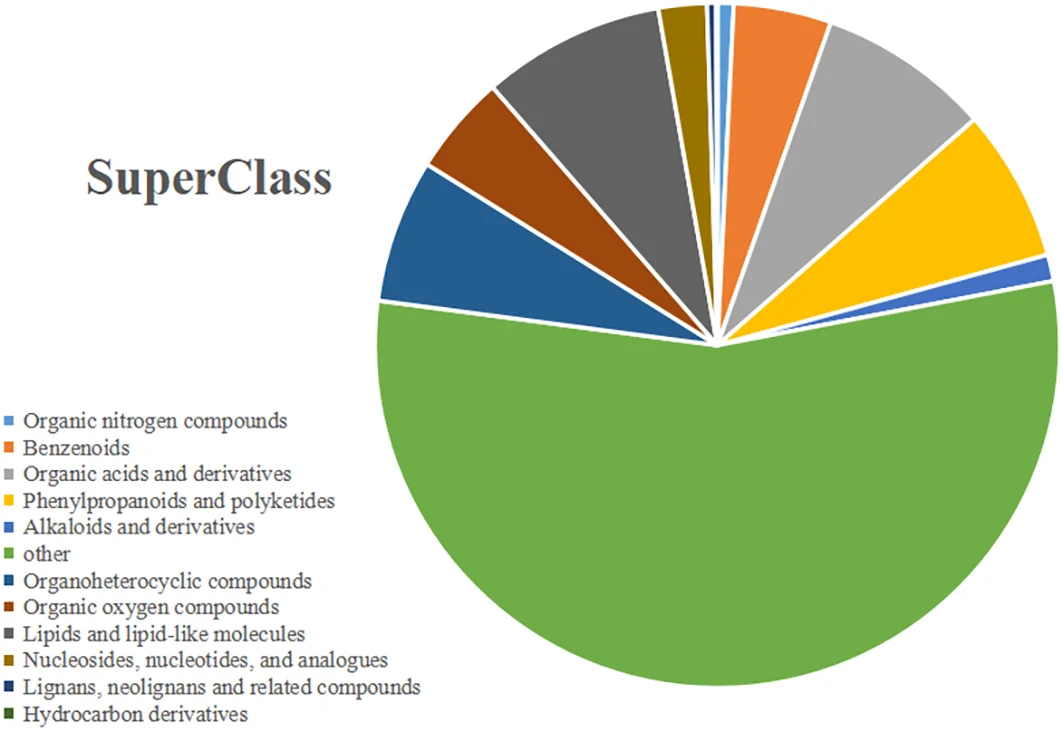
Pie chart depicting the categories of metabolites.

UHPLC-MS/MS-based non-targeted metabolomics was performed to detect differences in metabolite profiles of *P. cyrtonema* at different growth years. Princi*pal* component analysis (PCA) revealed that the samples within each group were highly reproducible, and the two groups were clearly separated (**Figure 6**), indicating substantial differences in their metabolite profiles. A total of 1,842 metabolites were annotated based on database matching, including 937 positive-ion-mode and 905 negative-ion-mode metabolites. Excluding the “Others” category, “Lipids and lipid-like molecules”, “Organic acids and derivatives”, “Phenylpropanoids and polyketides”, and “Organoheterocyclic compounds” were the top four most abundantly represented metabolite categories, containing 159, 150, 133, and 125 metabolites, respectively (**Figure 7**).

#### 3.2.2 Identification of differentially expressed metabolites

**Figure 8:**
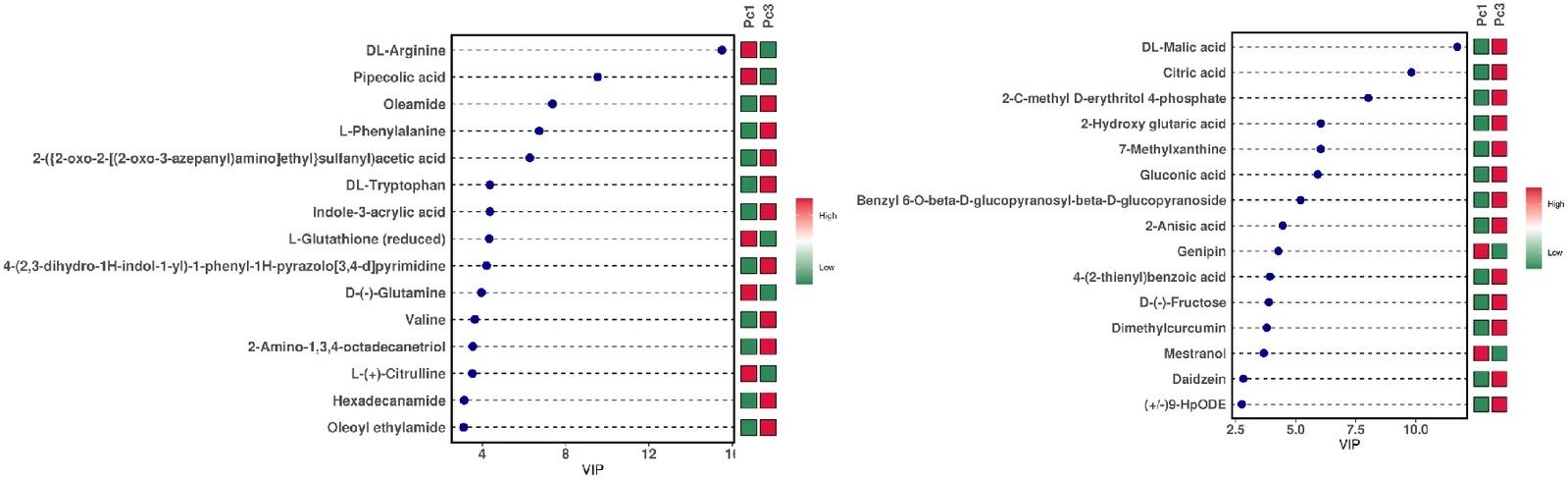
Top 15 differential metabolites in VIP index: the left is NEG mode and the right is POS mode.

The OPLS-DA model and Student’s t-test were applied to identify differentially expressed metabolites (DEMs), with p < 0.05 and VIP ≥ 1 as the criteria for significance. A total of 163 DEMs were identified, comprising 82 up-regulated and 81 down-regulated metabolites. Based on the classification of DEMs, the differences between up-regulated and down-regulated metabolites were mainly concentrated in “Organic acids and derivatives”, “Benzenoids”, and “Organic oxygen compounds” (**Table 4**). However, according to the comprehensive VIP index, the differences were also notable in “Lipids and lipid-like molecules”, “Organoheterocyclic compounds”, and “Alkaloids and derivatives” (**Figure 8**).

**Table 4:** Expression of differential metabolites of *P. cyrtonema*.

Superclass	Up-regulation	Down-regulation	All
Organic acids and derivatives	17	21	38
Benzenoids	7	2	9
Organoheterocyclic compounds	8	5	13
Lipids and lipid-like molecules	6	5	11
Alkaloids and derivatives	1	1	2
Organic oxygen compounds	3	10	13
Organic nitrogen compounds	0	1	1
Phenylpropanoids and polyketides	3	3	6
Nucleosides, nucleotides, and analogues	3	0	3
others	34	33	67

**Figure 9:**
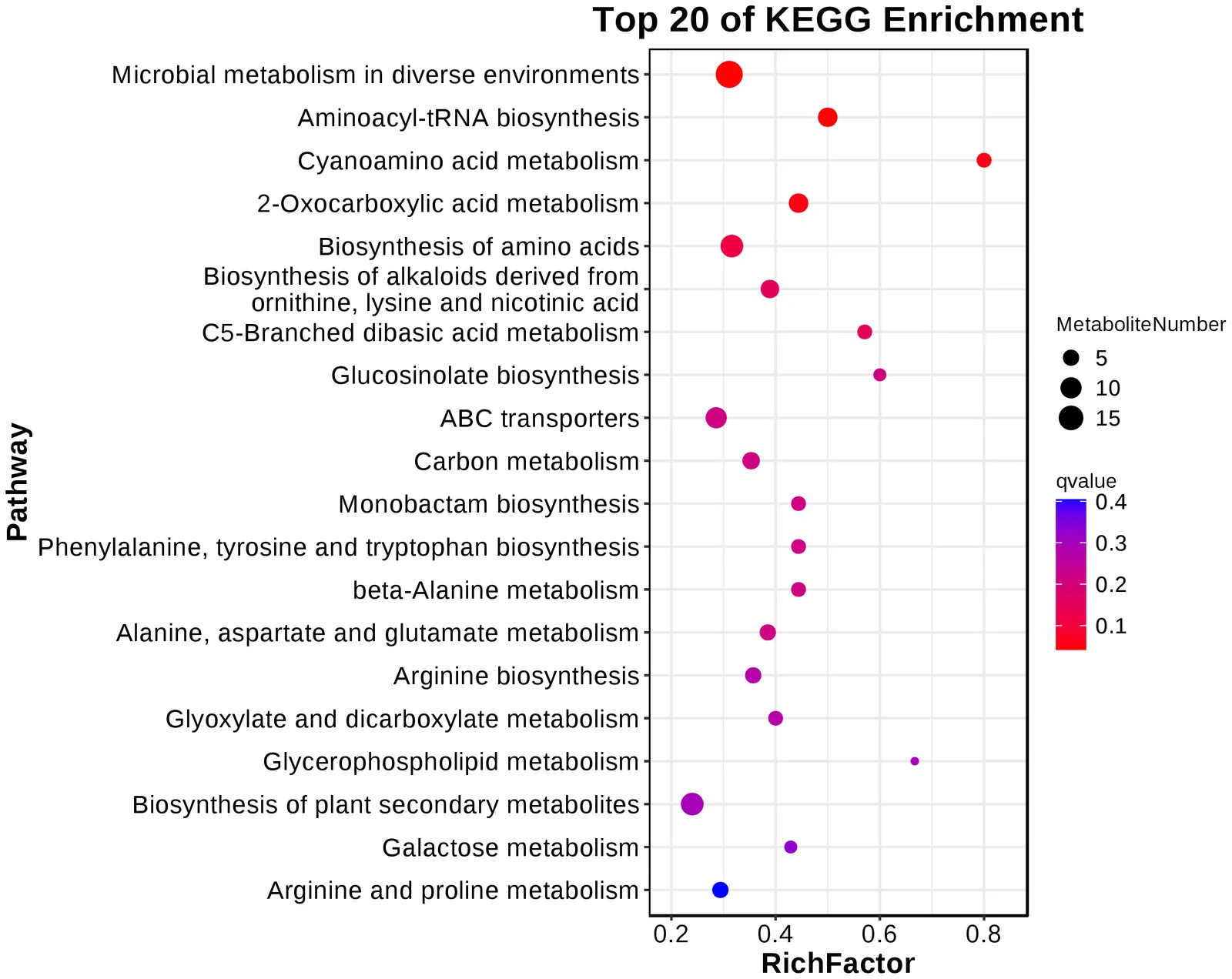
Top 20 KEGG enriched pathways of differential metabolites.

To understand the functional implications of these metabolite changes, we conducted KEGG pathway enrichment analysis of the DEMs. The significantly enriched pathways included microbial metabolism in diverse environments, aminoacyl-tRNA biosynthesis, cyanoamino acid metabolism, and biosynthesis of amino acids (**Figure 9**).

Of particular interest was the significant accumulation of L-phenylalanine, a central precursor in plant secondary metabolism. This finding, together with the co-enrichment of the phenylpropanoid biosynthesis pathway in the transcriptome data (**Figure 4**), suggests a potential coordinated enhancement of this pathway in three-year-old plants, which may provide the substrate for the biosynthesis of diverse phenolic compounds. However, we note that this interpretation is based on correlative evidence from the two omics datasets.

### 3.3 Integrated analysis of the transcriptome and metabolome

**Figure 10:**
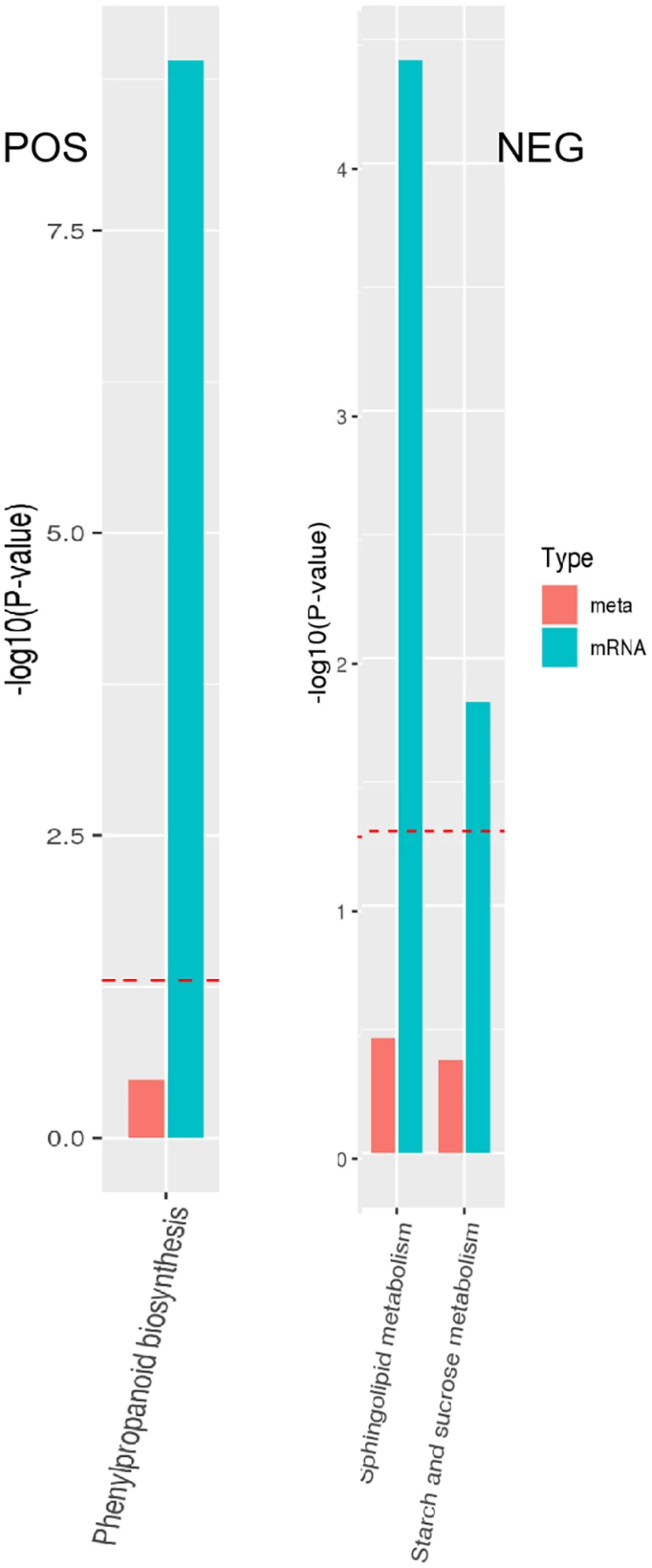
Related pathways co-enriched by transcriptomic and metabolomic differences.

**Figure 11:**
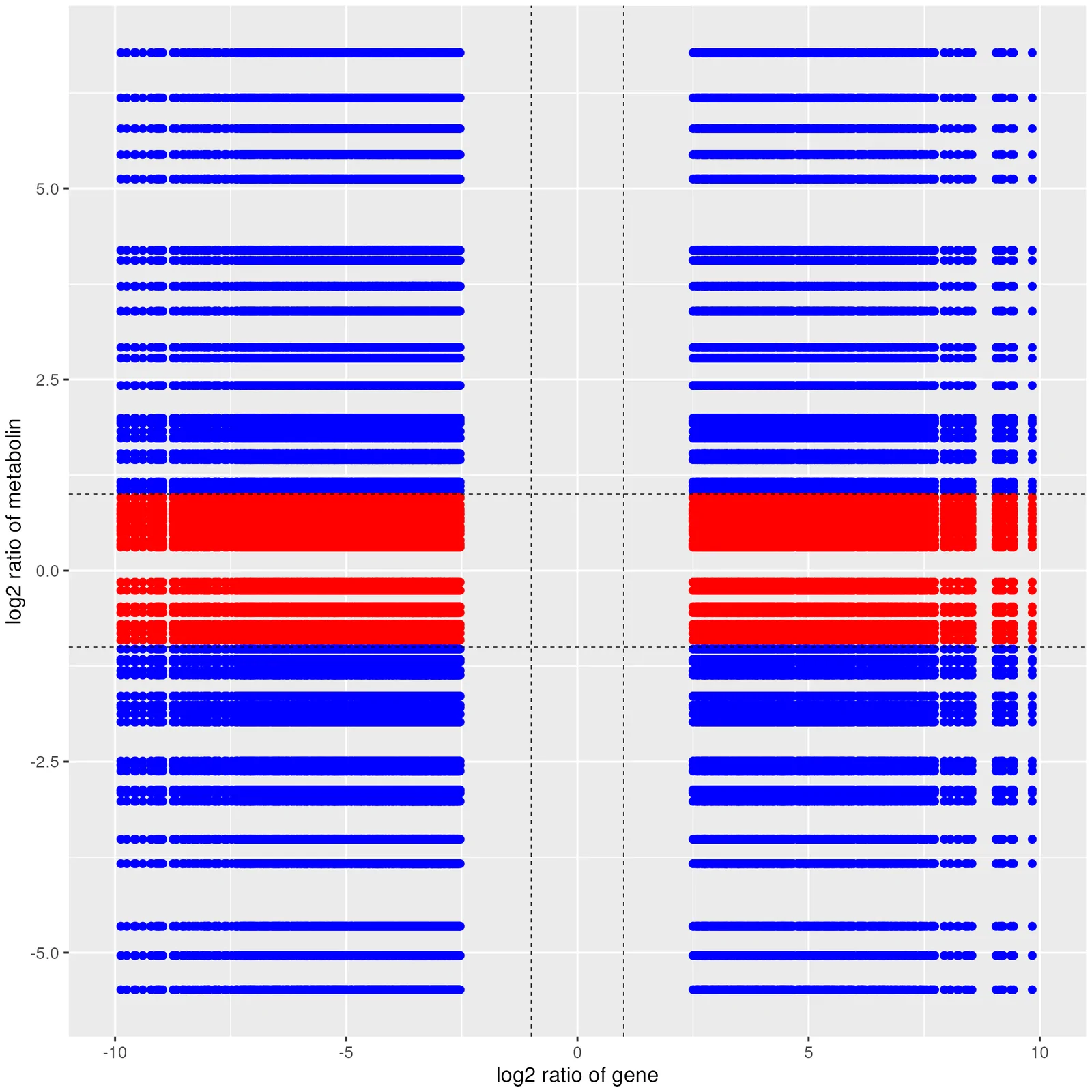
Nine-quadrant plots of metabolites against corresponding transcripts.

To further explore the relationship between gene expression and metabolite accumulation, the metabolic pathways in which DEGs and DEMs were jointly involved were analyzed. Among the co-enriched pathways, those potentially associated with growth regulation included sphingolipid metabolism, phenylpropanoid biosynthesis, and starch and sucrose metabolism (**Figure 10**). To more directly interrogate the relationship between transcriptional regulation and metabolic phenotypes, a correlation analysis between DEGs and DEMs was performed. As displayed in the nine-quadrant plot (**Figure 11**), strong positive correlations (Pearson correlation coefficient > 0.8) were observed between certain key metabolites and genes. Notably, the accumulation of L-phenylalanine in the rhizome was positively correlated with the expression of genes involved in the phenylpropanoid biosynthesis pathway. This correlation suggests a potential association between transcriptional changes in leaves and metabolic profiles in the rhizome, although we acknowledge that these are correlative observations and do not establish a direct causal relationship. Similarly, genes within the starch and sucrose metabolism pathway, including *SWEET14* and *β-fructofuranosidase*, formed distinct correlation clusters, suggesting their coordinated involvement in carbohydrate partitioning. In the sphingolipid metabolism pathway, key genes such as serine palmitoyltransferase (SPT) and PAL showed correlations with the differential metabolites trans-cinnamate 4-monooxygenase and *β-fructofuranosidase*.

### 3.4 Data availability

The complete lists of 1,957 differentially expressed genes and 163 differentially expressed metabolites identified in this study, including gene annotations, fold changes, p-values, metabolite identities, and VIP scores, are provided in **Supplementary Table 2** (Differential Gene Information) and **Supplementary Table 3** (Differential Metabolite Information).

## 4 Discussion

Our integrated analysis, which correlates the leaf transcriptome with the rhizome metabolome, provides a systems-level perspective on the growth and metabolic regulation in *P. cyrtonema*. This approach is grounded in the well-established “source–sink” theory in plant physiology, where photoassimilates and precursors synthesized in source leaves are transported to sink organs for growth and storage (Li et al., 2022). The significant correlations we observed between leaf DEGs and rhizome DEMs, particularly in pathways such as starch and sucrose metabolism and phenylpropanoid biosynthesis, suggest that transcriptional reprogramming in the leaves may be associated with metabolic changes in the developing rhizome. However, we emphasize that these associations are correlative in nature, and future studies involving direct rhizome transcriptomics and leaf metabolomics, combined with functional validation, would be needed to establish causal relationships.

It is generally considered that 1–3 years represents the optimal period for the accumulation of bioactive components in *P. cyrtonema*. Our integrated analysis of one-year-old and three-year-old plants suggests that the age-dependent growth regulation may primarily involve phenylpropanoid biosynthesis, sphingolipid metabolism, and starch and sucrose metabolism.

Regarding the phenylpropanoid pathway, the significant accumulation of L-phenylalanine and the upregulation of genes such as PAL in three-year-old rhizomes suggest that this precursor accumulation may not represent a metabolic endpoint but rather could indicate enhanced metabolic flux into the phenylpropanoid pathway. This interpretation is consistent with the established role of this pathway as a fundamental platform for generating diverse phenolic compounds crucial for structural integrity (e.g., through lignification) and defense in plants (Deng and Lu, 2017). We therefore propose that this metabolic shift may facilitate the synthesis of more complex phenolics, potentially contributing to the structural maturation and chemical defense of the aging rhizome. Similar metabolic adaptations have been reported in other medicinal plants where secondary metabolite accumulation is linked to growth and developmental stages (Zhou et al., 2025). This interpretation is further supported by evidence from model plants: genetic disruption of key enzymes such as cinnamate-4-hydroxylase (C4H) leads to severe developmental defects (Kim et al., 2021), underscoring the pathway’s indispensable role in normal plant growth—a principle that appears conserved in *P. cyrtonema*. The critical role of PAL is also illustrated by studies in other species; for instance, exogenous application of L-phenylalanine to *Brassica napus* significantly enhances phenolic compound expression and defense (Grey et al., 1997), and it improves vegetative growth in *Zea mays* L (Sarwar and Frankenberger, 1995). The conversion of phenylalanine to cinnamic acid, a precursor for many phenylpropanoids (Urban and Hura, 2023), is a key step in this process, as demonstrated in studies on sage (*Salvia officinalis* L.), where phenylalanine application improves essential oil quality (Rahmani Samani et al., 2019). Furthermore, disruption of downstream branches of this pathway, such as phloretin glycoside synthesis in apple, can significantly delay growth and reduce fruit size (Dare et al., 2017), while lignification of branch tissues enhances structural support and stress resistance (Wang et al., 2024). Therefore, the significant accumulation of L-phenylalanine in three-year-old *P. cyrtonema* may represent a metabolic adaptation for rhizome maturation and defense, although direct functional evidence is currently lacking.

Sphingolipids are widely distributed in eukaryotes and exhibit high diversity in plants, where they are not only involved in membrane structure but also participate in plant development regulation and stress responses (Huby et al., 2020; Liu et al., 2021). The first step of sphingolipid synthesis is catalyzed by serine *palmitoyltransferase* (SPT), which mediates the condensation of serine and palmitoyl-CoA (Haslam and Feussner, 2022). SPT, the rate-limiting enzyme of sphingolipid metabolism, is composed of two subunits, *LCB1* and *LCB2*. In *Arabidopsis thaliana*, partial RNAi silencing of *LCB1* results in reduced growth concomitant with downregulation of sphingolipid synthesis (Chen et al., 2006), while upregulation of *LCB2a1* in rice (*Oryza sativa*) enhances resistance to the brown planthopper (Begum et al., 2016). Orosomucoid (ORM) proteins function as negative regulators of SPT; plants with *ORM1*/2 interference exhibit premature senescence and formation of cell wall appositions, but their oxidative stress tolerance and defense capacity are also enhanced (Li et al., 2016). These findings suggest that upregulation of sphingolipid expression not only contributes to growth but also enhances plant defense. However, in the present study, the expression of L-serine and related enzymes in sphingolipid metabolism was found to be downregulated in three-year-old *P. cyrtonema* compared with one-year-old plants. We speculate that this may reflect the perennial growth habit of *P. cyrtonema*, which may increase sphingolipid synthesis during the first year by adopting a slower growth rate, thereby facilitating root system establishment. However, this hypothesis requires further experimental validation.

The medicinal part of *P. cyrtonema* is the rhizome, and the degree of rhizome development along with the content of bioactive metabolites are essential indicators for evaluating medicinal quality. The accumulation of polysaccharides in rhizomes is attributable to the efficiency of sucrose synthesis in leaves and its subsequent transport to sink tissues; polysaccharide content is a key determinant of both the medicinal quality of *P. cyrtonema* and its characteristic sweet taste (Zhao et al., 2025). In our previous studies, we found that *P. cyrtonema* rhizomes contain a large amount of fructose, a small amount of glucose, and trace amounts of other monosaccharides (Liu et al., 2022). In the starch and sucrose metabolism pathway, we observed significant expression of *β-fructofuranosidase*. Since fructose accumulation is closely associated with sucrose hydrolase activity, and *β-fructofuranosidase* is one such enzyme capable of hydrolyzing sucrose to fructose, this may promote fructose accumulation in the rhizome (Wang et al., 2019). Carbohydrate transport depends on sugar transporters, and the abundance of these transporters can influence the accumulation of organic matter and ultimately affect plant quality (Tian et al., 2024). The upregulation of the bidirectional sugar transporter *SWEET14* observed in this study is consistent with this notion. Although we did not detect significant accumulation of sugars in the combined analysis, the genes related to starch and sucrose metabolism were significantly altered, and the sucrose content in the rhizomes of three-year-old *P. cyrtonema* was decreased, which may provide a metabolic basis for the redistribution and accumulation of metabolites in older rhizomes.

### 4.1 Limitations of the study

Several limitations of this study should be acknowledged. First, all conclusions regarding gene–metabolite relationships are based on correlative evidence from transcriptomic and metabolomic co-enrichment and correlation analyses; no functional validation of the candidate genes (e.g., *SWEET14*, *β-fructofuranosidase, PAL, or SPT)* has been performed through genetic or biochemical approaches. Therefore, the specific roles of these genes in *P. cyrtonema* growth and development remain to be experimentally confirmed. Second, the transcriptome data were derived exclusively from leaf tissue, while the metabolome data were obtained from rhizome tissue. No direct rhizome transcriptome or leaf metabolome profiling was conducted; consequently, inferences regarding leaf-to-rhizome transport or causal regulatory relationships cannot be conclusively drawn from the current data alone. Complementary analyses, such as rhizome transcriptomics and leaf metabolomics, would be valuable in future studies to further validate the hypotheses generated here. Third, the statistical criteria employed for DEG identification (FDR < 0.05) and DEM identification (raw p < 0.05 with VIP ≥ 1) differ in their stringency, which may affect the comparability of the two omics datasets. Despite these limitations, we believe that our findings provide a useful foundation and generate testable hypotheses for future functional studies on the molecular mechanisms governing growth and metabolite accumulation in *P. cyrtonema*.

## 5 Conclusion

In this study, we investigated the leaf transcriptome and rhizome metabolome of one-year-old and three-year-old *P. cyrtonema*. Our integrated analysis suggests that several metabolic pathways and candidate genes may be associated with age-dependent growth regulation in *P. cyrtonema*. Specifically, the elevated abundance of L-phenylalanine in three-year-old rhizomes may be associated with enriched secondary metabolite diversity, potentially contributing to the metabolic basis for rhizome maturation and metabolite redistribution. Our data also suggest that the bidirectional sugar transporter *SWEET14* may facilitate sucrose transport to sink organs, and *β-fructofuranosidase* may hydrolyze sucrose to fructose for accumulation in the rhizome. In contrast, genes related to sphingolipid metabolism were found to be downregulated in older plants, which may reflect the perennial growth strategy of *P. cyrtonema*. We emphasize that all these findings are based on correlative transcriptomic and metabolomic data, and the causal roles of the identified candidate genes remain to be validated through future functional studies. This study provides data support and a theoretical foundation for further investigation into the molecular mechanisms underlying growth and metabolite accumulation in *P. cyrtonema*.

## Data Availability

The datasets presented in this study can be found in online repositories. The names of the repository/repositories and accession number(s) can be found below: https://www.ncbi.nlm.nih.gov/genbank/, SRR29139314.

## Supplementary material

Supplementary Table 1 Gene descriptions and primer sequences used for qRT-PCR.

Supplementary Table 2 Information table of differential genes.

Supplementary Table 3 Information table of differential metabolites.
