# Supplementary material for: Integrated transcriptome and metabolome analysis reveals key genes and metabolic pathways regulating growth and development in Polygonatum cyrtonema Hua
Source: Front Plant Sci. 2026 Sep 3;17:1940943. doi: 10.3389/fpls.2026.1940943 (PMC13582315; doi:10.3389/fpls.2026.1940943)
Supplement: Supplementary file 1 [file Table1.docx]

**Table S1** **Gene descriptions and primers used for qRT-PCR.**

| Genes | Amplicon Size （bp） | Primer pairs | TM value |
| --- | --- | --- | --- |
| TRINITY_DN21_c0_g1 | 92 | 5'-CTCCCCGGCCGAACTGACAA-3' | 61.6 |
|  |  | 5'-CCTTCTCGGACTTCGGCACCTT-3' | 60.4 |
| TRINITY_DN10270_c1_g1 | 89 | 5'-TTCAAAGGGGCACATCTAACTG-3' | 53.5 |
|  |  | 5'-ATATCAAACTCCGCTGCCACTT-3' | 54.5 |
| TRINITY_DN917_c0_g2 | 108 | 5'-TGGTGCGGGCGTTTCAGGT-3' | 60.8 |
|  |  | 5'-CTCGCCGCATTTGCAGTCAGAT-3' | 61.0 |
| TRINITY_DN2661_c0_g1 | 169 | 5'-GGAGGCGTTGCAAGCGAAAATA-3' | 60.1 |
|  |  | 5'-CATCAATGGCCACCGAAACAGG-3' | 60.9 |
| TRINITY_DN2150_c0_g3 | 153 | 5'-TCCCCCGGCAATTCTCCTTTAC-3' | 60.0 |
|  |  | 5'-TCGTGTTTGGCTTCCTTGATTC-3' | 55.7 |
| TRINITY_DN429_c1_g2 | 141 | 5'-CACCCGAAAGGCGAGGAGAATA-3' | 59.1 |
|  |  | 5'-CCGGACCGTGCCAGAATCAT-3' | 59.2 |
| TRINITY_DN1863_c0_g1 | 150 | 5'-CGGCGAAGCAGAGGAGGATTT-3' | 59.3 |
|  |  | 5'- CGATAGGCCATTGCACCAGTTG-3' | 59.0 |
| TRINITY_DN1568_c1_g1 | 199 | 5'-CAAGCAGGCAGATGTGGTGAT-3' | 54.9 |
|  |  | 5'-GATAGCCCGTCGGATTTTAGC-3' | 54.6 |
| TRINITY_DN798_c0_g1 | 108 | 5'-TTCGAGTCCGGACAAGAGTTCA-3' | 56.2 |
|  |  | 5'-GCGCGGGTTTATCAATCCA-3' | 55.7 |
| TRINITY_DN2280_c1_g1 | 140 | 5'-CAAACGGTCGGAGAACAAGAAG-3' | 55.4 |
|  |  | 5'-TGGCCTCAGTCGCATCGCTACC-3' | 63 |
| 18S | 156 | 5'-CAACCATAAACGATGCCGA-3' | 52.8 |
|  |  | 5'-AGCCTTGCGACCATACTCC-3' | 57.7 |
